# Predictive accuracy of a general-purpose artificial intelligence model for cut-out following proximal femoral nailing

**DOI:** 10.1007/s00402-026-06447-5

**Published:** 2026-08-01

**Authors:** Guy Ben Arie, Yaniv Warschawski, Nissan Amzallag, Hadar Gan-Or, Tomer Ben-Tov, Amal Khoury, Idit Horev, Nadav Graif, Guy Morag

**Affiliations:** 1 Tel Aviv University, Tel Aviv, Israel; 2 Tel Aviv Sourasky Medical Center, Tel Aviv, Israel; 3 Hillel Yaffe Medical Center, Hadera, Israel

**Keywords:** ChatGPT, Large language model, Predictive accuracy, Cephalomedullary nail, Intertrochanteric fracture, Calibration

## Abstract

**Introduction:**

Cut-out is the most consequential mechanical complication after proximal femoral nailing and requires prompt recognition. General-purpose artificial intelligence (AI) models with image-interpretation capability are increasingly accessible, yet their diagnostic performance for this task is unknown. We evaluated ChatGPT’s accuracy for predicting cut-out following proximal femoral nailing and compared TFNA and Gamma nail subgroups.

**Materials and methods:**

In this retrospective predictive-accuracy study, 989 patients (683 TFNA, 306 Gamma nail; mean age 83.8 years; cut-out prevalence 2.5%) were analysed. For each case, three baseline radiographs — the injury anteroposterior and lateral views and the immediate postoperative control radiograph, all obtained before any complication could be radiographically present — together with the full clinical data set (excluding complication-related information) were submitted to ChatGPT, which returned a binary prediction (yes/no) and probability estimate (0–100%). The follow-up radiographs on which cut-out becomes apparent were not provided to the model. Ground truth was established on subsequent follow-up radiographs by a departmental review board requiring agreement of two senior surgeons. Accuracy metrics were calculated with Wilson 95% confidence intervals (CI); exact McNemar’s and Mann-Whitney U tests assessed directional bias and calibration. Reporting followed STARD guidelines.

**Results:**

ChatGPT showed a sensitivity of 68.0% (95% CI 48.4–82.8%), specificity of 62.7% (59.6–65.7%), PPV of 4.5% (2.8–7.1%), NPV of 98.7% (97.4–99.3%), and AUC of 0.694 (0.580–0.790). Performance was higher for TFNA (sensitivity 73.7%, AUC 0.730) than for Gamma nail (sensitivity 50.0%, AUC 0.606). The model over-predicted cut-out, with 360 false positives against 8 false negatives (45:1; McNemar’s *p* < 0.001, Holm-corrected). Calibration was inverted: the median predicted probability was lower for correct (9%) than for incorrect (40%) classifications (Mann-Whitney *p* < 0.001).

**Conclusions:**

ChatGPT demonstrated limited, clinically insufficient accuracy for predicting cut-out following proximal femoral nailing, with a prohibitive false-positive burden and inverted calibration. Subgroup point estimates were lower for Gamma nail cases, but this exploratory difference did not reach statistical significance. General-purpose AI models are not currently suitable as a substitute for, or adjunct to, surgeon-led surveillance of this complication. Task-specific training, external validation, and defined clinical boundaries are required before such tools can be considered for fracture follow-up pathways.

## Introduction

Proximal femoral nailing is the most commonly performed surgical procedure for intertrochanteric and subtrochanteric hip fractures, which represent a leading cause of morbidity and mortality in the elderly population [1–3]. Cut-out, defined as the migration of the cephalic fixation device through the femoral head, is the most frequent and clinically consequential mechanical complication of this procedure, with reported incidence rates of approximately 1–8% depending on fracture pattern, implant type, and reduction quality [4–6]. Prompt recognition of developing cut-out on follow-up radiographs is essential, as early revision surgery — before complete implant perforation and acetabular destruction — is associated with substantially better outcomes than delayed intervention [7, 8].

The emergence of general-purpose artificial intelligence (AI) models with multimodal image-interpretation capability has introduced a new category of tools that are directly accessible to clinicians, researchers, and patients without requiring dedicated validation as medical devices. Unlike task-specific AI systems designed and trained for a single diagnostic task, large language models such as ChatGPT can accept radiographic images alongside free-text prompts and produce structured outputs including binary diagnoses and probabilistic estimates. This accessibility has outpaced the evidence base for their clinical performance [9, 10].

The diagnostic accuracy of dedicated AI systems for fracture detection and orthopaedic imaging analysis has been increasingly studied, with promising results reported across multiple subspecialties [11–16]. Systematic reviews and meta-analyses have documented that task-specific AI models frequently achieve sensitivity and specificity above 90% in controlled testing environments for narrow detection tasks such as fracture identification, implant recognition, and radiographic loosening assessment [11–13]. However, the performance of general-purpose multimodal models for the more demanding task of complication identification — requiring recognition of subtle implant-bone relationships and biomechanical failure patterns on follow-up radiographs — has not been formally characterised. Furthermore, the reliability of AI-generated probability estimates as a guide to clinical decision-making has received little attention in the orthopaedic literature, and concerns about overoptimistic performance estimates, limited algorithmic transparency, and insufficient calibration assessment remain significant barriers to clinical translation [16–18].

The primary aim of this study was to determine the predictive accuracy of ChatGPT for cut-out following proximal femoral nailing, including sensitivity, specificity, PPV, NPV, and AUC. Secondary aims were to compare performance between the TFNA nail (DePuy Synthes) and the Gamma nail (Stryker) to characterise the directionality of classification errors, to evaluate the internal consistency between ChatGPT’s binary predictions and its continuous probability outputs, and to assess the calibration of its probability estimates relative to actual classification correctness.

## Materials and methods

### Study design and cohort

We performed a retrospective predictive accuracy study of patients who underwent proximal femoral nailing at our institution between January 2014 and January 2026. The study was approved by the institutional Helsinki (research ethics) review board, approval no. XXXX; patient consent was waived given the retrospective design and anonymised data handling. The study was performed in accordance with the 1964 Declaration of Helsinki and its later amendments.

The primary clinical question motivating this study was whether a general-purpose AI model could predict subsequent cut-out from the injury and immediate postoperative radiographs, before the complication was radiographically apparent. All diagnostic accuracy metrics reflect conditions that would be considered for use in genuine clinical surveillance practice.

**Fig. 1:**
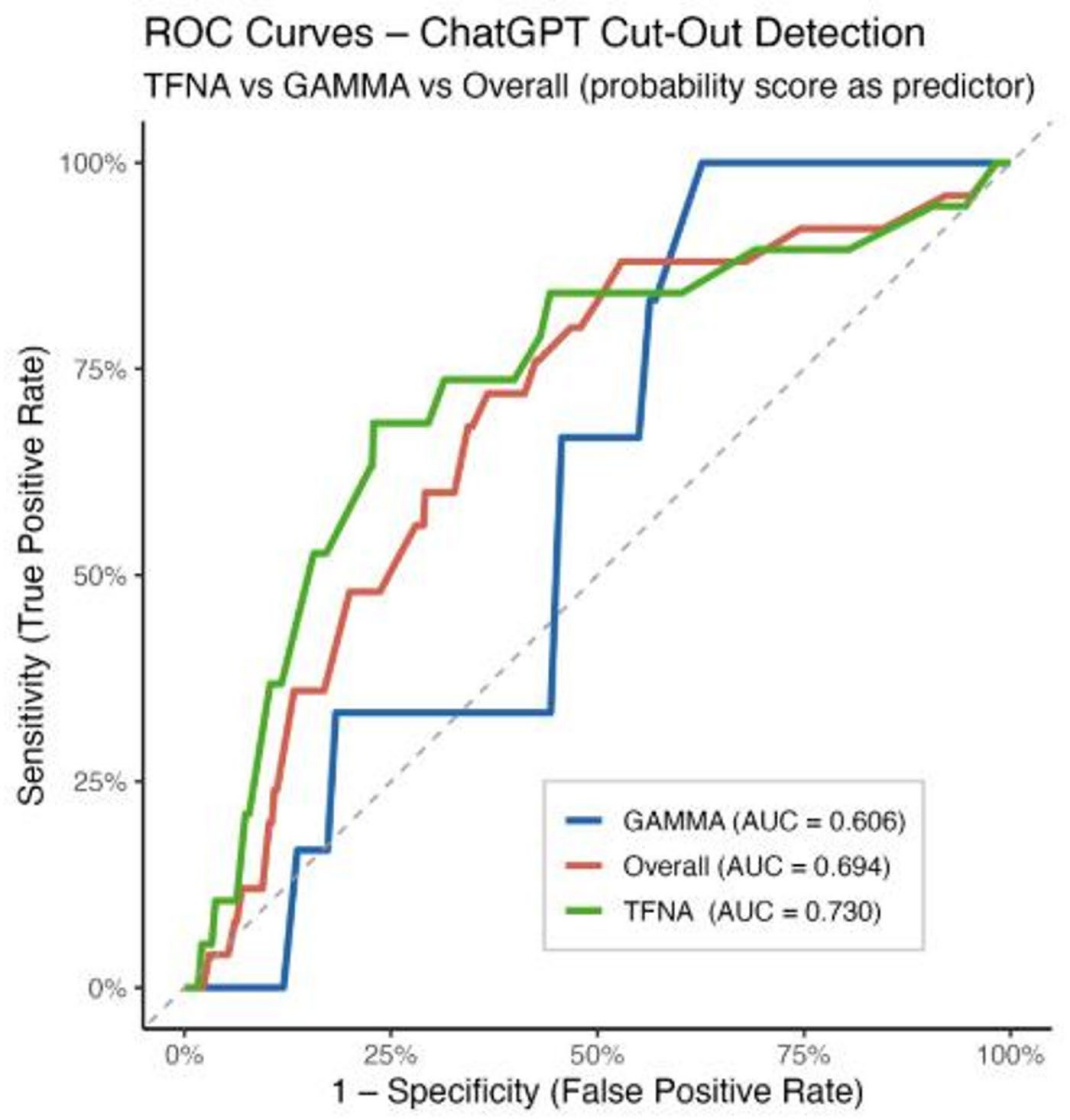
Receiver Operating Characteristic (ROC) Curves for ChatGPT Cut-Out Prediction — Overall, TFNA, and Gamma Nail Subgroups. ROC curves generated using the ChatGPT-assigned continuous probability score (0–100%) as the predictor variable, with ground truth cut-out status as the binary outcome. The area under the curve (AUC) is presented for the overall cohort (AUC = 0.694; 95% CI 0.580–0.790), the TFNA subgroup (AUC = 0.730; 95% CI 0.592–0.845), and the Gamma nail subgroup (AUC = 0.606; 95% CI 0.444–0.783). The diagonal dashed line represents chance-level discrimination (AUC = 0.50). AUC confidence intervals derived from 2,000-iteration bootstrap resampling. TFNA = Trochanteric Fixation Nail Advanced; AUC = area under the receiver operating characteristic curve

Inclusion criteria were: (1) TFNA or Gamma nail proximal femoral nail for an intertrochanteric or subtrochanteric hip fracture; (2) availability of injury anteroposterior and lateral radiographs, an immediate postoperative control radiograph, and at least one subsequent follow-up radiograph for outcome adjudication. Exclusion criteria were: pathological fracture, periprosthetic fracture, prior ipsilateral hip surgery, inadequate radiographic quality, or incomplete data. After application of these criteria, 989 patients were included in the final analytic cohort (683 TFNA, 306 Gamma nail) Fig. 1.

## Clinical and radiological determination of cut-out

**Fig. 2:**
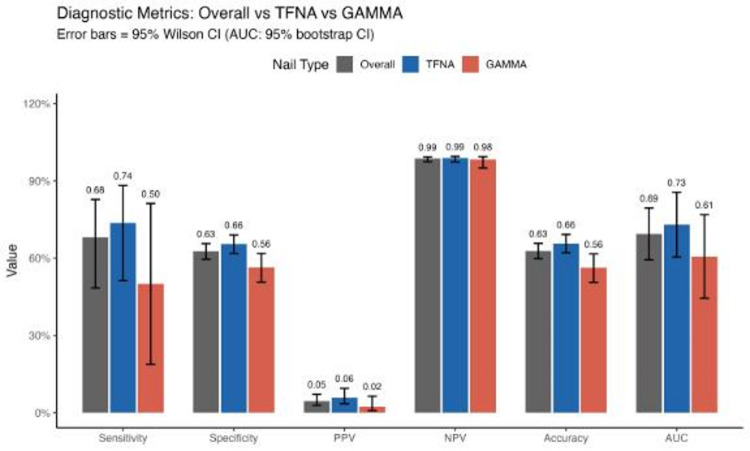
Predictive Performance Metrics for ChatGPT Cut-Out Identification — Overall Cohort, TFNA, and Gamma Nail Subgroups. Grouped bar chart displaying six primary predictive accuracy metrics for the overall cohort (grey), TFNA subgroup (blue), and Gamma nail subgroup (red). Error bars represent 95% Wilson confidence intervals for sensitivity, specificity, positive predictive value (PPV), negative predictive value (NPV), and accuracy; the 95% CI for the area under the receiver operating characteristic curve (AUC) was derived from 2,000-iteration bootstrap resampling. Values above bars indicate point estimates. Note the uniformly low PPV across all groups, reflecting the low prevalence of cut-out (2.5%) in this surveillance cohort. TFNA = Trochanteric Fixation Nail Advanced; PPV = positive predictive value; NPV = negative predictive value; AUC = area under the receiver operating characteristic curve

All patients underwent routine postoperative radiographic surveillance for a minimum of 11 months, except where precluded by earlier death. Imaging was performed per institutional protocol and was not triggered by clinical suspicion of cut-out; the cohort therefore represents a consecutive unselected routine surveillance population rather than a selected high-risk or symptomatic group. Cut-out — defined as migration or perforation of the cephalic fixation device relative to its immediate postoperative position — was diagnosed on the follow-up radiographs by direct side-by-side comparison with the immediate postoperative baseline film. Diagnosis was established through the institutional orthopaedic departmental review board, a formal clinical governance process at which cases are presented by the original operating surgeon and reviewed by a minimum of two senior orthopaedic surgeons; agreement between reviewers was required, and cases with disagreement were discussed until consensus was reached. Critically, the follow-up radiographs used to establish the reference standard were not among the images provided to ChatGPT (see below), ensuring that the ground truth was determined independently of the model’s inputs. Tip-apex distance was assessed qualitatively during adjudication, but individual numeric values were not systematically retained in the analytic dataset; Cleveland zone position was likewise not formally recorded Fig. 2.

## ChatGPT evaluation protocol

For each case, three radiographic images were submitted simultaneously to ChatGPT in a single query: the injury anteroposterior and lateral radiographs and the immediate postoperative control radiograph (typically obtained on postoperative day 1–3). No follow-up radiographs were provided. This input was selected to replicate the information available to a treating clinician at the immediate postoperative point — before any cut-out could be radiographically present — so that the model’s task was to predict subsequent cut-out rather than to detect an already-visible finding.

In addition to the radiographic images, each submission included the complete available clinical data for that patient, comprising: nail type (TFNA or Gamma nail), blade or screw fixation subtype where applicable, nail length, fracture classification (AO/OTA) [19], laterality, patient age, sex, BMI, ASA physical status classification, and the number of days from surgery to the postoperative radiograph. Crucially, this clinical data package deliberately excluded any information directly related to the complication itself — specifically, no prior diagnosis of cut-out, no previous revision surgery, no clinical suspicion, and no outcome data were disclosed. ChatGPT therefore received the same contextual clinical information a treating surgeon would have at the immediate postoperative assessment, without knowledge of the outcome under investigation. This approach was chosen to reflect real-world clinical conditions whilst isolating ChatGPT’s radiographic interpretation capability from outcome-guided bias.

**Fig. 3:**
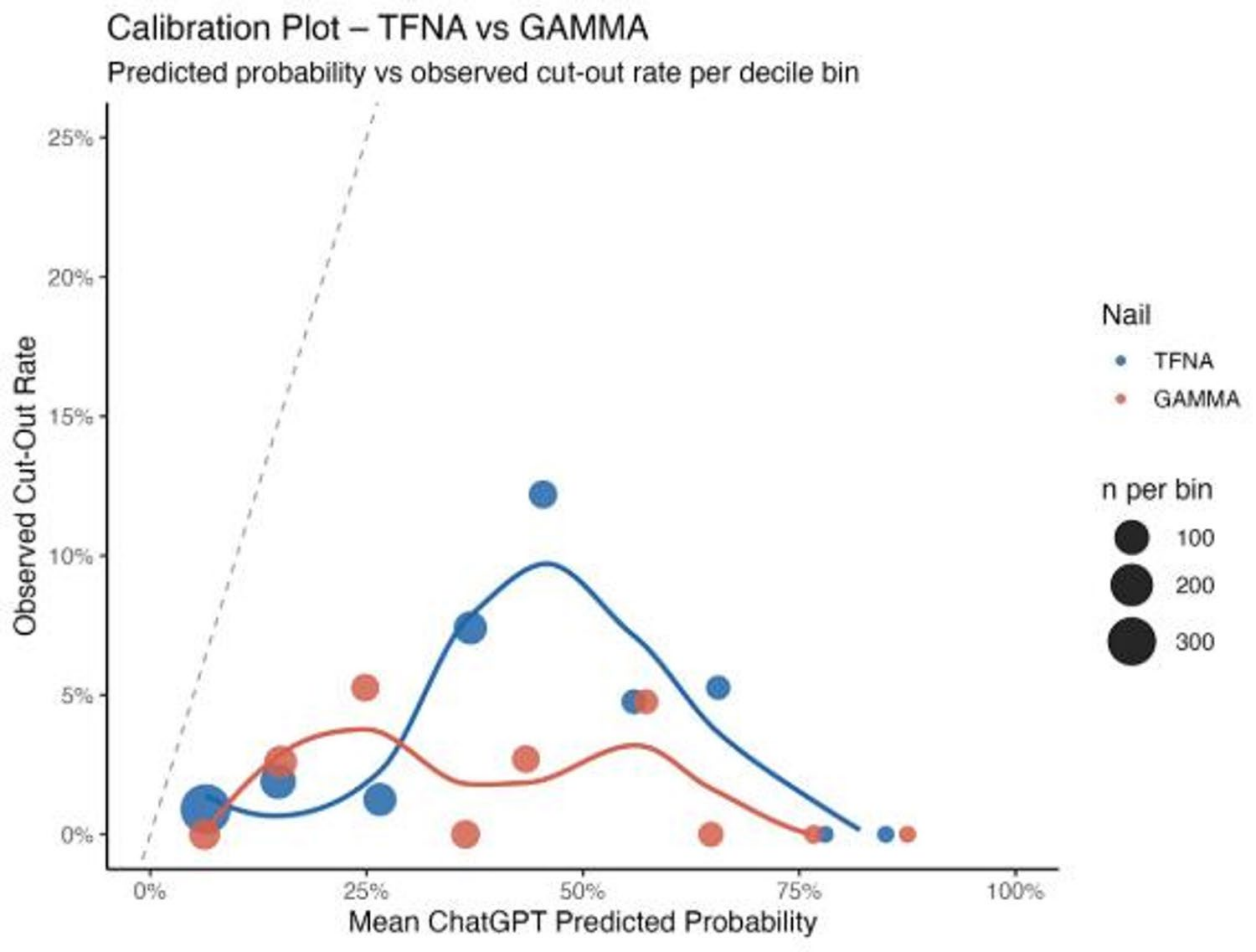
Calibration Plot — ChatGPT Predicted Cut-Out Probability versus Observed Cut-Out Rate by Nail Type. Calibration plot displaying the observed cut-out rate (y-axis) against the mean ChatGPT-predicted probability (x-axis) per decile bin, stratified by nail type (TFNA, blue; Gamma nail, red). Point size is proportional to the number of patients in each bin. The diagonal dashed line represents perfect calibration (predicted probability equals observed rate). LOESS smoothing curves are shown for each nail type. For a well-calibrated model, points should cluster along the diagonal; deviation above the diagonal indicates underestimation and deviation below indicates overestimation. The observed pattern — in which the highest observed cut-out rates correspond to mid-range rather than high predicted probabilities, and points at high predicted probabilities have near-zero observed rates — demonstrates inverted calibration, confirming that ChatGPT’s probability estimates are not reliable indicators of true cut-out risk in this cohort. TFNA = Trochanteric Fixation Nail Advanced; LOESS = locally estimated scatterplot smoothing

Submissions were made to GPT-5.3 through the web interface between September 2025 and March 2026. A single standardised prompt was pre-specified and used without modification for every case: “Based on the anteroposterior and lateral radiographs obtained at the time of injury and the postoperative control radiograph, and considering the following clinical parameters — patient age, BMI, sex, ASA classification, nail type, nail length, fracture classification, and number of days from surgery to the postoperative radiograph — will this patient develop cut-out? Answer yes or no only, and provide a probability estimate as a percentage.”. Each case was submitted in a new, independent session to prevent carryover from prior responses. No patient identifiers were included. ChatGPT’s binary prediction (yes/no) and continuous probability estimate (0–100%) were recorded verbatim Fig.3.

### Statistical analysis

Statistical analysis was performed in R (R Foundation for Statistical Computing, Vienna, Austria). For all diagnostic accuracy proportions, we calculated Wilson 95% confidence intervals, which are more reliable than Wald intervals at low prevalence. The area under the receiver operating characteristic curve (AUC) was calculated using the continuous ChatGPT probability score as the predictor, with 2,000-iteration bootstrap confidence intervals. The Brier score was used as a composite measure of probabilistic calibration. Diagnostic performance was compared between TFNA and Gamma nail subgroups using Fisher’s exact test for proportions and a bootstrap permutation test for AUC comparison.

The directional asymmetry between false-positive and false-negative errors was assessed within each group using exact paired McNemar’s tests, with Holm correction applied across the family of six prespecified comparisons to control the family-wise error rate. The Youden’s J index (sensitivity + specificity − 1) and F1-score were calculated as summary measures of discriminative performance. To assess whether ChatGPT’s probability estimates reflected classification correctness — analogous to the confidence calibration analyses used in recent AI characterisation studies [9, 10, 20] — predicted probability was compared between correctly and incorrectly classified cases using the Mann-Whitney U test, with results reported as median (interquartile range). A threshold sensitivity analysis was performed at 40%, 50%, and 70% probability cut-points to examine the trade-off between sensitivity and specificity across alternative classification thresholds. This study was reported in accordance with the Standards for Reporting of Diagnostic Accuracy Studies (STARD) guidelines [21].

## Results

### Patient characteristics

The final analytic cohort comprised 989 patients (mean age 83.8 years [SD 7.9]; 666 women [67.3%]; mean follow-up 11.1 months [SD 17.6]). The TFNA group included 683 patients (mean age 83.6 years [SD 8.2]; 449 women [65.7%]; cut-out prevalence 2.8%), and the Gamma nail group included 306 patients (mean age 84.5 years [SD 7.3]; 217 women [70.9%]; cut-out prevalence 2.0%). Full baseline characteristics are presented in Table 1. The TFNA group had a higher proportion of 31-A3 fractures (11.6% vs. 5.9%) and a lower proportion of subtrochanteric fractures (9.1% vs. 15.0%), with overall fracture distribution differing significantly between groups (*p* < 0.001). ASA grade III or above was present in 68.8% of the TFNA group compared with 52.4% of the Gamma nail group. Blade fixation was used in 556 TFNA cases (81.4%) and screw fixation in 127 (18.6%); all Gamma nail fixations used the standard lag screw. Short nails (≤ 170 mm TFNA; ≤180 mm Gamma) were the most common configuration in both groups (68.2% and 72.9%, respectively). Follow-up duration was substantially longer in the Gamma nail group (mean 22.8 months [SD 28.3]) than in the TFNA group (mean 6.4 months [SD 5.5]; *p* < 0.001), reflecting historical differences in institutional follow-up protocols for the two implant types during the study period. Twenty-five cut-out cases were identified across the primary analytic cohort: 19 in the TFNA group (2.8%) and 6 in the Gamma nail group (2.0%; *p* = 0.588). Detailed characteristics of cut-out cases, including timing and implant details, are presented in Table 2.

**Table 1:** Patient Demographics and Implant Characteristics by Nail Type (*n* = 989)

Variable	Overall (*n* = 989)	TFNA (*n* = 683)	Gamma Nail (*n* = 306)	*p*-Value*
*Demographics*				
Mean age, years (SD)	83.8 (7.9)	83.6 (8.2)	84.5 (7.3)	0.079
Female sex, n (%)	666 (67.3%)	449 (65.7%)	217 (70.9%)	0.126
Mean BMI, kg/m² (SD)	25.1 (8.1)	25.3 (8.9)	24.6 (4.4)	0.321
*ASA Physical Status*,* n (%)*				
I	17 (1.7%)	10 (1.5%)	7 (2.3%)	< 0.001
II	340 (34.4%)	201 (29.4%)	139 (45.4%)	< 0.001
III	568 (57.4%)	427 (62.5%)	141 (46.1%)	< 0.001
IV	60 (6.1%)	43 (6.3%)	17 (5.6%)	< 0.001
V	4 (0.4%)	2 (0.3%)	2 (0.7%)	< 0.001
*Fracture Classification (AO/OTA)*,* n (%)*				
31-A1	211 (21.3%)	166 (24.3%)	45 (14.7%)	< 0.001
31-A2	573 (57.9%)	376 (55.1%)	197 (64.4%)	< 0.001
31-A3	97 (9.8%)	79 (11.6%)	18 (5.9%)	< 0.001
Subtrochanteric	108 (10.9%)	62 (9.1%)	46 (15.0%)	< 0.001
*Implant Characteristics*				
Side: left, n (%)	501 (50.7%)	350 (51.2%)	151 (49.3%)	0.614
Short nail†, n (%)	689 (69.7%)	466 (68.2%)	223 (72.9%)	0.295
Intermediate nail†, n (%)	213 (21.5%)	156 (22.8%)	57 (18.6%)	0.295
Long nail†, n (%)	87 (8.8%)	61 (8.9%)	26 (8.5%)	0.295
Blade fixation, n (%)	556 (56.2%)†	556 (81.4%)	N/A	—
Screw fixation, n (%)	127 (12.8%)†	127 (18.6%)	N/A	—
*Clinical Outcomes*				
Mean follow-up, months (SD)	11.1 (17.6)	6.4 (5.5)	22.8 (28.3)	< 0.001
In-hospital / 30-day mortality, n (%)	383 (38.7%)	150 (22.0%)	233 (76.1%)	< 0.001
Mean time to death, months (SD)	23.4 (26.5)	6.5 (6.1)	34.3 (29.0)	< 0.001
Cut-out, n (%)	25 (2.5%)	19 (2.8%)	6 (2.0%)	0.588

*SD* standard deviation. * p-Values compare TFNA vs. Gamma nail subgroups: independent-samples t-test for continuous variables; chi-square test for ASA and fracture classification; Fisher’s exact test for binary variables. † Nail-length categories: TFNA — short ≤ 170 mm, intermediate 175–235 mm, long ≥ 260 mm; Gamma nail — short ≤ 180 mm, intermediate 200–260 mm, long ≥ 300 mm. Blade/screw overall percentages are of the total cohort (*n* = 989); subgroup percentages are of TFNA cases only (*n* = 683). N/A = not applicable (Gamma nail uses a standard lag screw integral to the implant design). ASA = American Society of Anesthesiologists physical status classification. [INSERT] = value not calculable from available data and requires author insertion before submission.

**Table 2:** Characteristics and Timing of Cut-Out Cases (*n* = 25)

Variable	TFNA (*n* = 19)	Gamma Nail (*n* = 6)	*p*-Value
*Patient Demographics*			
Mean age, years (SD)	85.4 (7.5)	78.8 (6.9)	0.064
Female sex, n (%)	15 (78.9%)	4 (66.7%)	0.606
Mean BMI, kg/m² (SD)	25.5 (5.9)	23.1 (6.9)	0.803
*Fracture Classification (AO/OTA)*,* n (%)*			
31-A1	1 (5.3%)	0 (0.0%)	0.194
31-A2	7 (36.8%)	5 (83.3%)	
31-A3	11 (57.9%)	1 (16.7%)	
*Implant Details*			
Blade fixation, n (%)	16 (84.2%)	N/A	—
Screw fixation, n (%)	3 (15.8%)	N/A	—
Short nail, n (%)	10 (52.6%)	4 (66.7%)	0.476
Intermediate nail, n (%)	8 (42.1%)	1 (16.7%)	
Long nail, n (%)	1 (5.3%)	1 (16.7%)	
*Cut-Out Timing*			
Median time to cut-out, days (IQR)	62.5 (44–101)	78 (52–238)	0.443
Mean time to cut-out, days (SD)	97 (142)	225 (312)	
Range, days	2–647	29–831	—
*ChatGPT Performance on True Cut-Out Cases*			
Correctly identified (true positive), n (%)	14 (73.7%)	3 (50.0%)	0.344
Missed (false negative), n (%)	5 (26.3%)	3 (50.0%)	0.344
Median ChatGPT predicted probability, %	40%	28%	0.873

*SD* standard deviation; *IQR*   interquartile range; *AO/OTA*  Arbeitsgemeinschaft für Osteosynthesefragen/Orthopaedic Trauma Association; *N/A*  not applicable. p-Values compare TFNA versus Gamma nail subgroups: Mann-Whitney U test for continuous and time-to-event variables; Fisher’s exact test for binary categorical variables, with the Freeman–Halton extension for the three-category fracture-classification and nail-length comparisons (a single p-value reported for the overall distribution). Nail-length categories: short < 200 mm, intermediate 200–299 mm, long ≥ 300 mm. Blade versus screw fixation applies to the TFNA group only; the Gamma nail incorporates an integral lag screw (N/A). Mean BMI was unavailable for one Gamma nail case (BMI denominator *n* = 5). Time to cut-out was unavailable for one TFNA case (timing denominator *n* = 18). Surgical management and time-to-revision data are not included, as the underlying revision procedure and revision-date fields were unavailable in the analytic dataset.

### Overall diagnostic performance

Across the entire cohort, ChatGPT correctly predicted 17 of 25 true cut-out cases, yielding a sensitivity of 68.0% (95% CI 48.4–82.8%). It correctly predicted 604 of 964 cases without cut-out, giving a specificity of 62.7% (95% CI 59.6–65.7%). The PPV was 4.5% (95% CI 2.8–7.1%), reflecting the low prevalence of the complication and the large number of false-positive predictions. The NPV was 98.7% (95% CI 97.4–99.3%). Overall accuracy was 62.8% (95% CI 59.7–65.7%). The AUC for the continuous probability score was 0.694 (95% CI 0.580–0.790), the Brier score was 0.093, the F1-score was 0.085, and Youden’s J index was 0.307. Full diagnostic accuracy metrics with 95% confidence intervals are presented in Table 3.

**Table 3:** Prediction Accuracy of ChatGPT for Cut-Out Following Proximal Femoral Nailing

Metric	Overall (*n* = 989)	TFNA (*n* = 683)	Gamma Nail (*n* = 306)	p*
*Confusion Matrix*				
True positives (TP)	17	14	3	—
False positives (FP)	360	229	131	—
False negatives (FN)	8	5	3	—
True negatives (TN)	604	435	169	—
*Primary Prediction Accuracy Metrics*				
Sensitivity	68.0%	73.7%	50.0%	0.342
*95% CI*	*[48.4–82.8%]*	*[51.2–88.2%]*	*[18.8–81.2%]*	
Specificity	62.7%	65.5%	56.3%	0.014
*95% CI*	*[59.6–65.7%]*	*[61.8–69.0%]*	*[50.7–61.8%]*	
Positive Predictive Value (PPV)	4.5%	5.8%	2.2%	0.156
*95% CI*	*[2.8–7.1%]*	*[3.5–9.4%]*	*[0.8–6.4%]*	
Negative Predictive Value (NPV)	98.7%	98.9%	98.3%	0.580
*95% CI*	*[97.4–99.3%]*	*[97.4–99.5%]*	*[95.0–99.4%]*	
Overall Accuracy	62.8%	65.7%	56.2%	0.007
*95% CI*	*[59.7–65.7%]*	*[62.1–69.2%]*	*[50.6–61.7%]*	
*Secondary Metrics*				
AUC (ROC curve)	0.694	0.730	0.606	0.240
*95% CI*	*[0.580–0.790]*	*[0.592–0.845]*	*[0.444–0.783]*	
F1-Score	0.085	0.107	0.043	—
Youden’s J Index	0.307	0.392	0.063	—
Brier Score†	0.093	0.077	0.129	—
*Confidence Calibration*				
Median predicted probability — correct classifications	9%	8%	14%	—
Median predicted probability — incorrect classifications	40%	35%	42%	—
Mann-Whitney U p-value (correct vs. incorrect)	< 0.001	< 0.001	< 0.001	—
*Directional Bias*				
FP : FN ratio	45 : 1	45.8 : 1	43.7 : 1	—
McNemar’s test p-value (Holm-corrected)	< 0.001	< 0.001	< 0.001	—

*AUC* area under the receiver operating characteristic curve; *PPV *positive predictive value; *NPV*  negative predictive value; *FP*  false positive; *FN* false negative; *TP*  true positive; *TN *  true negative. 95% confidence intervals (CI) calculated using the Wilson score method for sensitivity, specificity, PPV, NPV, and accuracy; 2,000-iteration bootstrap for AUC. * p-Values compare TFNA vs. Gamma nail subgroups: Fisher’s exact test for proportions; bootstrap permutation test for AUC. † Brier score: lower values indicate better probabilistic calibration; 0 = perfect, prevalence-based null = 0.024. McNemar’s test evaluates the asymmetry between false-positive and false-negative counts within each group; Holm correction applied across the family of six prespecified comparisons. Confidence calibration p-values derived from Mann-Whitney U test comparing predicted probability between correctly and incorrectlyclassified cases.TFNA = Trochanteric Fixation Nail Advanced; PPV = positive predictive value; NPV = negative predictive value; AUC = area under the receiver operating characteristic curve

### Performance by nail type

In the TFNA subgroup (*n* = 683), ChatGPT achieved a sensitivity of 73.7% (95% CI 51.2–88.2%), specificity of 65.5% (95% CI 61.8–69.0%), PPV of 5.8% (95% CI 3.5–9.4%), NPV of 98.9% (95% CI 97.4–99.5%), and overall accuracy of 65.7% (95% CI 62.1–69.2%). The AUC was 0.730 (95% CI 0.592–0.845) and the Brier score was 0.077, representing the strongest discriminative and calibration performance across all subgroups. Youden’s J index was 0.392.

Performance was substantially inferior in the Gamma nail subgroup (*n* = 306). Sensitivity was 50.0% (95% CI 18.8–81.2%), specificity 56.3% (95% CI 50.7–61.8%), PPV 2.2% (95% CI 0.8–6.4%), NPV 98.3% (95% CI 95.0–99.4%), and overall accuracy 56.2% (95% CI 50.6–61.7%). The AUC was 0.606 (95% CI 0.444–0.783), the Brier score was 0.129, and Youden’s J was 0.063. These Gamma nail estimates rest on only six true cut-out events and carry wide confidence intervals; they should be regarded as exploratory and hypothesis-generating. The difference in AUC between TFNA and Gamma nail subgroups was 0.124 (95% CI − 0.105 to 0.327; *p* = 0.240), which did not reach statistical significance, likely due to the small number of true cut-out events available for analysis.

### Directional error bias

ChatGPT demonstrated a highly significant and consistent directional bias toward over-prediction of cut-out across all groups. Overall, 360 false positives were recorded against only 8 false negatives (FP: FN ratio 45:1; McNemar’s *p* < 0.001, Holm-corrected). This asymmetry was similarly pronounced in the TFNA subgroup (229 FP vs. 5 FN; 45.8:1; *p* < 0.001) and the Gamma nail subgroup (131 FP vs. 3 FN; 43.7:1; *p* < 0.001). The direction of this bias is toward caution — ChatGPT was systematically more likely to raise a false alarm than to miss a true cut-out — though the magnitude of over-calling rendered this behaviour clinically impractical.

### Threshold sensitivity analysis

To determine whether alternative probability thresholds could improve diagnostic performance, we applied cut-points of 40%, 50%, and 70% to ChatGPT’s continuous probability score and compared the resulting metrics with the default binary classification output. Results across all thresholds and both nail-type subgroups are presented in Table 4.

**Table 4:** Threshold Sensitivity Analysis — Predictive Performance of ChatGPT Across Alternative Probability Cut-Points (Overall Cohort, *n* = 989) Positive cut-out classification defined as ChatGPT-assigned probability ≥ stated threshold. Default = ChatGPT-generated binary yes/no output

Metric (Overall Cohort, *n* = 989)	Default ChatGPT Y/*N*	40% Threshold†	50% Threshold	70% Threshold
*Confusion Matrix*				
True positives (TP)	17	12	5	0
False positives (FP)	360	193	99	18
False negatives (FN)	8	13	20	25
True negatives (TN)	604	771	865	946
*Primary Predicitve Accuracy Metrics*				
Sensitivity	68.0%	**48.0%**	20.0%	0.0%
*95% CI*	*[48.4–82.8%]*	*[30.0–66.5%]*	*[8.9–39.1%]*	*[0.0–13.3%]*
Specificity	62.7%	**80.0%**	89.7%	98.1%
*95% CI*	*[59.6–65.7%]*	*[77.3–82.4%]*	*[87.7–91.5%]*	*[97.1–98.8%]*
PPV	4.5%	**5.9%**	4.8%	0.0%
*95% CI*	*[2.8–7.1%]*	*[3.4–10.0%]*	*[2.1–10.8%]*	*[0.0–17.6%]*
NPV	98.7%	**98.3%**	97.7%	97.4%
*95% CI*	*[97.4–99.3%]*	*[97.2–99.0%]*	*[96.5–98.5%]*	*[96.2–98.3%]*
Overall Accuracy	62.8%	**79.2%**	88.0%	95.7%
*95% CI*	*[59.7–65.7%]*	*[76.5–81.6%]*	*[85.8–89.8%]*	*[94.2–96.8%]*
*Secondary Metrics*				
AUC (ROC curve)‡	0.694	0.694	0.694	0.694
F1-Score	0.085	**0.104**	0.078	0.000
Youden’s J Index	0.307	**0.280**	0.097	−0.019
Brier Score‡	0.093	0.093	0.093	0.093
*Directional Bias*				
FP : FN ratio	45 : 1	15 : 1	5 : 1	—
McNemar’s p (Holm-corrected)	< 0.001	< 0.001	< 0.001	0.286
*TFNA Subgroup (n = 683)*				
Sensitivity	73.7%	52.6%	21.1%	0.0%
Specificity	65.5%	84.3%	92.6%	98.6%
Youden’s J	0.392	0.370	0.137	−0.014
*Gamma Nail Subgroup (n = 306)*				
Sensitivity	50.0%	33.3%	16.7%	0.0%
Specificity	56.3%	70.3%	83.3%	97.0%
Youden’s J	0.063	0.037	0.000	−0.030

*AUC* area under the receiver operating characteristic curve; *PPV* positive predictive value; *NPV *negative predictive value; *FP* false positive; *FN* false negative. 95% CIs calculated using the Wilson score method. † Shaded column (40% threshold) represents the threshold yielding the highest Youden’s J index and F1-score of any alternative threshold tested, and is highlighted for reference. ‡ AUC and Brier score are properties of the continuous probability score and are invariant to threshold selection; they remain constant across all threshold configurations. The default ChatGPT Y/N classification applies an internal threshold estimated at approximately 25–35%, inferred from observed discordance between binary predictions and continuous probability outputs (of 377 binary-positive cases, only 104 received probability ≥ 50% and only 18 received probability ≥ 70%). Youden’s J = Sensitivity + Specificity − 1; values ≤ 0 indicate performance at or below chance. McNemar’s test evaluates FP versus FN asymmetry within each threshold; Holm correction applied across the family of threshold comparisons. TFNA = Trochanteric Fixation Nail Advanced; PPV = positive predictive value; NPV = negative predictive value; AUC = area under the receiver operating characteristic curve

The default ChatGPT binary prediction — which applies an internal threshold estimated to approximate 25–35% based on observed discordance between binary and probability outputs — achieved the highest sensitivity (68.0%; 95% CI 48.4–82.8%) of any configuration tested. Raising the threshold to 40% reduced sensitivity to 48.0% (95% CI 30.0–66.5%) whilst improving specificity to 80.0% (95% CI 77.3–82.4%). At 40%, Youden’s J index was 0.280 and the F1-score was 0.104 — the highest values achieved by any alternative threshold — indicating that this represents the best attainable balance between sensitivity and specificity when the continuous probability score is used as the classifier. Nonetheless, a sensitivity of 48% implies that more than half of all true cut-outs would be missed, which is clinically unacceptable for a complication surveillance tool.

At a 50% threshold, sensitivity fell to 20.0% (95% CI 8.9–39.1%), meaning 4 in every 5 true cut-outs would be missed, while specificity rose to 89.7% (95% CI 87.7–91.5%). Youden’s J was 0.097, reflecting near-negligible net discriminative value. At a 70% threshold, ChatGPT identified zero true cut-out cases across the entire cohort (sensitivity 0.0%; 95% CI 0.0–13.3%), rendering the model entirely non-functional as a prediction tool at this cut-point, despite achieving a specificity of 98.1%. McNemar’s test for directional bias remained significant at all thresholds below 70% (all Holm-corrected *p* < 0.001); at 70% it was no longer significant (*p* = 0.286), reflecting near-complete cessation of positive predictions rather than genuine improvement in classification accuracy. In both nail-type subgroups, the threshold trajectory was consistent: sensitivity declined monotonically with increasing threshold while specificity improved, but at no threshold did both metrics simultaneously achieve clinically acceptable values. In the Gamma nail subgroup, Youden’s J fell to zero at a 50% threshold and became negative at 70%, confirming chance-level or sub-chance performance. The AUC (0.694) and Brier score (0.093) remained invariant across all threshold configurations, representing the ceiling of discriminative and calibrative performance achievable by any threshold-based decision rule applied to ChatGPT’s probability output.

### Internal consistency of binary and probability outputs

A notable internal inconsistency was identified between ChatGPT’s binary predictions and its continuous probability estimates. Of the 377 cases classified as cut-out by the binary output, only 104 (27.6%) received a predicted probability of 50% or greater, and only 18 (4.8%) received a probability of 70% or greater. This indicates that ChatGPT frequently assigned a positive binary classification while simultaneously expressing low or moderate probabilistic confidence — a pattern inconsistent with the behaviour of a coherent probabilistic classifier.

### Confidence calibration

Confidence calibration was inverted relative to expected behaviour. The median ChatGPT-predicted cut-out probability was significantly lower for correctly classified cases (median 9%) than for incorrectly classified cases (median 40%; Mann-Whitney U, *p* < 0.001). This pattern was consistent across both nail-type subgroups (TFNA: median 8% correct vs. 35% incorrect; Gamma nail: median 14% correct vs. 42% incorrect; both *p* < 0.001). These findings indicate that ChatGPT’s probability estimate could not be used as a reliable guide to the correctness of its classification output — and that higher predicted probabilities were, counterintuitively, associated with a greater likelihood of error.

## Discussion

This study provides a comprehensive assessment of the accuracy of a general-purpose AI model — ChatGPT — for the prediction of cut-out following proximal femoral nailing. Using a retrospective cohort of 989 patients across two nail types, we demonstrate that ChatGPT’s performance is limited and clinically insufficient for standalone use in this predictive task. The key findings are four-fold: (1) overall discriminative performance was modest (AUC 0.694), with sensitivity of only 68% and specificity of 63%; (2) subgroup point estimates were lower for the Gamma nail than for the TFNA (AUC 0.606 vs. 0.730), although this exploratory difference did not reach statistical significance and rests on only six Gamma nail events; (3) the model exhibited a systematic and quantitatively large bias toward over-prediction of cut-out, generating 45 false positives for every false negative; and (4) confidence calibration was inverted — the model expressed higher predicted probabilities precisely when it was making errors.

The modest overall AUC of 0.694 is consistent with the performance ceiling that has been observed when general-purpose AI models are applied to specialised orthopaedic imaging tasks requiring narrow domain expertise [16–18]. Unlike dedicated AI systems trained on large, task-specific datasets for fracture detection or implant surveillance — which have demonstrated substantially higher discriminative performance in controlled evaluation settings [11–15] — ChatGPT was not developed or validated for this purpose. Its ability to identify cut-out relies on general pattern recognition capabilities rather than optimised feature extraction for the subtle radiographic signs — implant position relative to the femoral head, tip-apex distance changes, cortical breach — that characterise this complication [4, 5]. The finding that AUC was numerically higher for TFNA (0.730) than for Gamma nail (0.606) — a difference that did not reach statistical significance (*p* = 0.240) — raises the hypothesis that ChatGPT may perform better on implant configurations whose radiographic appearance is more represented in its training data, or whose cut-out morphology is more visually distinctive. This difference warrants further investigation but should be interpreted cautiously given the small number of true cut-out events, particularly in the Gamma nail group.

The magnitude of the false-positive burden is the most clinically important finding. A PPV of 4.5% means that approximately 95 in every 100 cases flagged by ChatGPT as probable cut-out would not represent true cut-out. In a surveillance programme applied to a high-volume arthroplasty and fracture fixation service, this would generate a prohibitive volume of unnecessary clinical re-assessment, additional imaging, and patient anxiety. While the directional bias toward over-calling is arguably preferable to the alternative missing a true cut-out, the practical consequence of a 45:1 FP: FN ratio is that the signal is lost in noise. A clinician acting on ChatGPT’s binary output would find it clinically inactionable.

The real-world clinical benchmark provided by this cohort places these figures in sharp relief. Cut-out in this study was detected by the original operating surgeon during the course of routine follow-up review — a clinician with complete knowledge of the patient, the intraoperative findings, the implant configuration, and the clinical trajectory, reviewing scheduled postoperative radiographs over a mean follow-up of 12 months. That process identified 25 true cut-out cases across 989 patients with, by definition, a false-positive rate of zero — no unnecessary revision or additional imaging was triggered by a spurious cut-out prediction. ChatGPT, working only from the baseline injury and immediate postoperative radiographs and the same structured clinical data — and never seeing the follow-up films on which the surgeon made the diagnosis — generated 360 false positives against 17 true positives. This is not a head-to-head blinded comparison, and it should not be presented as one; the surgeon and ChatGPT were operating under different conditions and at different points in the clinical pathway. It is, however, a direct and clinically meaningful contextualisation: the human surveillance process that this study asks whether AI could replicate or augment performed, in this cohort, with a qualitative superiority that the quantitative metrics make difficult to overstate.

This comparison also clarifies the study’s clinical question. The aim was not to evaluate ChatGPT as a radiologist adjunct in a formal diagnostic workup — where some false-positive burden may be an acceptable trade-off for sensitivity — but to assess whether it could support the routine surgeon-led surveillance that represents standard postoperative care. Against that benchmark, its performance falls substantially short.

The threshold analysis (Table 4) further illuminates the nature of ChatGPT’s failure mode in this task. The trajectory across thresholds — monotonically rising specificity at the cost of catastrophically falling sensitivity — is the characteristic signature of a model that lacks sufficient discriminative signal to achieve simultaneous adequacy in both dimensions. A well-performing model with an AUC substantially above 0.694 would display a flatter, more favourable sensitivity-specificity trade-off across the threshold range, with a broad region where both metrics remain clinically useful. In our data, no such region exists. By 50%, sensitivity has fallen below the level of usefulness; by 70%, it has reached zero.

The behaviour at the 70% threshold deserves particular comment. A specificity of 98.1% at this cut-point might superficially appear as a strength — the model almost never fires a false positive. However, this is achieved by the near-complete suppression of all positive predictions: only 18 cases in the entire cohort of 989 received a probability of 70% or above, and none of them were true cut-outs. The model has, in effect, shut down as a prediction tool. A test that never returns a positive result has no diagnostic utility regardless of its apparent specificity, and Youden’s J of − 0.019 at this threshold falling below zero — confirms that performance has formally deteriorated below chance.

The threshold analysis also provides additional evidence for the internal incoherence between ChatGPT’s binary and probability outputs. The default binary classification, which yielded the best sensitivity of any configuration, was effectively applying a threshold of approximately 25–35% — far below what a clinician would typically regard as the boundary between a positive and negative prediction. The fact that this low internal threshold outperforms every higher alternative underscores that ChatGPT’s probability scale is not anchored to conventional probabilistic meaning in this clinical context. A reported probability of 30% was, in this model’s output, functionally equivalent to a positive classification. Clinicians interpreting these outputs without awareness of this calibration failure risk systematic misunderstanding of the model’s predictions.

Taken together, the threshold analysis and the confidence calibration findings converge on the same conclusion: the limitation of ChatGPT in this task is not a correctable parameter but a structural property of the model’s discriminative capacity. Neither threshold adjustment nor probability re-scaling can compensate for an AUC ceiling of 0.694. The clinical implication is direct — improving ChatGPT’s performance for this specific task would require fundamental changes to the model or its training, not adjustments to how its existing output is interpreted.

The finding of inverted confidence calibration extends and parallels observations made in recent characterisation studies of general-purpose AI models applied to orthopaedic imaging tasks [9, 10, 20]. In our cohort, ChatGPT assigned higher predicted probabilities to its incorrect predictions than to its correct ones. The mechanistic explanation is straightforward in the context of a low-prevalence condition: the majority of correct classifications were true negatives, to which ChatGPT appropriately assigned low cut-out probabilities. The errors, however, were predominantly false positives — cases to which ChatGPT assigned substantial predicted probabilities despite the absence of cut-out. The result is that a clinician using the probability output as a decision aid would be systematically misled: a probability of 40% or above, which appears clinically meaningful, was in our cohort more characteristic of a false alarm than a true positive.

The provision of full clinical data alongside three radiographic views warrants specific discussion in the context of the observed results. The decision to include patient demographics, nail type, fixation subtype, nail length, and fracture classification was deliberate — it was designed to replicate the information available to a treating clinician at the immediate postoperative assessment, and to ensure that any ChatGPT performance deficit could not be attributed to a lack of clinical context. Crucially, all complication-related information was withheld; ChatGPT was not informed whether cut-out had previously been suspected, diagnosed, or treated. The systematic over-prediction therefore occurred despite access to the same rich multiparameter clinical context available to a human reviewer, including the implant type most predictive of cut-out risk. This cannot be explained by informational disadvantage; that ChatGPT’s lower Gamma nail performance persisted despite receiving nail-specific data suggests the failure lies in radiographic pattern integration rather than clinical context deprivation. Critically, this over-prediction occurred in a routine surveillance cohort — a population specifically selected because it was not enriched for clinical suspicion — which means the false-positive burden observed here is likely to be representative of, rather than an underestimate of, the burden that would be encountered in real-world deployment.

Several limitations of this study warrant acknowledgement. First, this was a single-centre retrospective study, and the findings may not generalise to other institutions, patient populations, or imaging protocols. Second, a single standardised prompt and a single model version were used, reflecting the resource constraints of a manually conducted study at this scale. This means that the results characterise the performance of one prompt applied to one model at one point in time, and that prompt sensitivity analysis, multi-model comparison, and longitudinal re-evaluation across model versions were outside the scope of this study. We note, however, that a single realistic prompt is arguably more representative of actual clinical use than an optimised multi-prompt ensemble, since a clinician deploying ChatGPT in practice would apply one prompt, not a grid-searched composite. Systematic prompt engineering and multi-model evaluation are natural priorities for future work. Third, as general-purpose AI models are updated continuously, performance at the time of this study cannot be assumed to be stable across future versions; periodic re-evaluation would be necessary before any proposed clinical application [9, 10]. The majority of published AI models in orthopaedics are derived from retrospective, single-centre datasets and lack robust external validation, and the present study shares these limitations [16, 18]. Fourth, the comparison between TFNA and Gamma nail subgroups should be considered exploratory: with only six true cut-out events in the Gamma nail group, the subgroup confidence intervals are wide and the AUC comparison was not statistically significant. Fifth, the study did not include a formal blinded human reader comparison; the real-world surgeon detection performance described in this cohort provides a contextual benchmark but should not be interpreted as a formally controlled head-to-head evaluation.

On the basis of these findings, we do not support the standalone clinical use of ChatGPT or equivalent general-purpose AI models for the prediction of cut-out following proximal femoral nailing. For such a tool to approach clinical utility in this context, it would require substantially higher sensitivity — ideally exceeding 90% — alongside a PPV sufficient to generate an actionable signal above the background rate of clinical suspicion. The NPV of 98.7% is notable and suggests a potential role for rule-out in very-low-risk surveillance scenarios, though this requires prospective validation. Future development in this area should focus on task-specific model training using curated orthopaedic implant datasets, quantitative integration of established predictive variables such as the tip-apex distance and Cleveland zone mapping, and formal external validation in diverse patient cohorts before any clinical deployment is considered [16–18, 22]. Progress toward safe adoption of AI tools in orthopaedic surveillance will require development of models using real-world, multicenter data; rigorous, transparent reporting of methodology, calibration, and bias; and prospective evaluation across heterogeneous patient populations and imaging protocols [16, 18, 23, 24].

## Conclusion

These results indicate that general-purpose AI models are not currently suitable as a substitute for or standalone adjunct to surgeon-led surveillance for this complication, and that the path to clinical utility requires task-specific training, rigorous external validation, and defined operational boundaries.

## Data Availability

No datasets were generated or analysed during the current study.
